# Male Survivors of Institutional Child Sexual Abuse: A Review

**DOI:** 10.1177/15248380241277272

**Published:** 2024-09-20

**Authors:** Paul Wyles, Patrick O’Leary, Menka Tsantefski, Amy Young

**Affiliations:** 1 Griffith University, Gold Coast, QLD, Australia; 2 Southern Cross University, Lismore, NSW, Australia

**Keywords:** child sexual abuse, CSA, institutions, male sexual abuse, maltreatment, prevalence, survivors, trauma, victims

## Abstract

Male child sexual abuse is over-represented in institutional settings. This realization has increasingly come into public focus in recent decades initially through lived experience, often with male survivors’ stories told in the media and subsequently through court cases and government inquiries. Beginning at the turn of the century with the Irish Commission to Inquire into Child Abuse (1999–2009), numerous national and state inquiries into institutional child abuse followed around the world. This scoping review asks the question: What is known from the research about the institutional child sexual abuse of males? Conducted in 2023 five databases were used (APA PsycINFO, CINAL, Medline, Scopus, Web of Science) producing 973 studies for screening. Applying the Arksey and O’Malley framework resulted in 29 studies meeting the inclusion criteria, which were analyzed. Of the 29 studies, 27 could be categorized into 3 broad areas of focus: survivor experience, impact, and disclosure. Two further studies considered: turning points and meaning making. The findings are discussed under the following headings: disclosure, impacts (emotional, mental health, alcohol, and other impacts), and what is helpful to victim/survivors. Implications for practice, policy, and research are examined along with limitations of the current research.

## Introduction

There is a large and growing research literature on child sexual abuse (CSA),^1^ which is predominantly focused on females with less focus on the CSA of males, resulting in gaps in the research literature (Gill & Tutty, 1999; Rapsey et al., 2020; Trask et al., 2010) and subsequently only a modest appreciation of the male specific impacts of CSA and treatment responses (O’Gorman et al., 2023). What we know about the effects of CSA on females is not necessarily always applicable to males (Hartill, 2005). One group of male survivors of CSA are those whose abuse occurred in an institutional environment. Conscious of the over-representation of males in institutional CSA, this scoping review purposely focuses on research that engaged/examined adult males regarding the experience and the aftermath of institutional CSA. The significance of this study is its locating of the male experience of CSA and trauma in institutional contexts; asking the research question: *What is known from the research about the institutional child sexual abuse of males?*

The abuse of children was identified in the literature (Kempe et al., 1962) in the early 1960s. In the 1970s, victim/survivors,^2^ feminists, and social workers in women and children’s shelters and hospitals brought the issue to public attention and demanded action (Cossins, 2000; Herman, 1992; Rush, 1981). Before the 1970s, it was generally believed that CSA was rare and, where it existed, was in disadvantaged groups, or those from particular racial backgrounds and, in almost all cases, it was believed to only affect girls (Hunter, 1990; Pleck, 2004; Whittier, 2009). Most research in this field has addressed CSA by examining the prevalence, prevention, impacts, treatments, and perpetrators. Much of the research in this area lacks a victim orientation (Shon & Tewksbury, 2021), and there is a dearth of longitudinal research (Walsh et al., 2010). Too little attention has been paid to the sexual abuse of male children (Hartill, 2009; Ressel et al., 2018) or to institutional CSA (Sprober et al., 2014), resulting in evidence gaps (Independent Inquiry into Child Sexual Abuse, 2017).

Many jurisdictions around the world have been slow to recognize rape of boys or men in law and in some cases still do not acknowledge it. It was only in 1994 in the United Kingdom that male rape was recognized instead of it being a case of “buggery” which carried significantly less penalties (Javaid, 2014). There is increasing and strong evidence that most victim/survivors of institutional CSA are male (Romano & De Luca, 2001; Royal Commission into Institutional Responses into Child Sexual Abuse, 2017a), especially in religious institutions (John Jay College of Criminal Justice, 2004; Parkinson & Cashmore, 2017). All these factors both perceptually and structurally have impeded focus being given to male victim/survivors of CSA, particularly where it has occurred in an institutional context.

### Institutional CSA

While girls are more likely to be abused by a male family member, boys experience extrafamilial abuse “. . . in the offender’s home, institution or in a public place” (Cashmore & Shackel, 2014, p. 77) and . . . “Boys . . . (are) twice as likely as girls to experience CSA by institutional caregivers” (Mathews et al., 2024, p. 6). Explanations for higher prevalence of institutional male CSA include the higher numbers of males in institutional care such as boarding schools, residential care, and youth detention (Sprober et al., 2014), and the opportunities churches historically provided for abusers to access boys (Parkinson et al., 2012). The Royal Commission into Institutional Responses into Child Sexual Abuse (2017b) met with 6,875 individuals in private sessions between May 2013 and May 2017 where 64.3% were male victim/survivors. In Spain, high rates of male survivors have been identified in religious institutions, with one study reporting the rate at 81% (Tamarit et al., 2023). Contemporary research in Australia has shown that males remain more likely to be abused in institutional settings, particularly religious institutions compared to females. Specifically, Hunt et al. (2024) found Australians aged over 16 had experienced CSA by a leader or other adult within a religious organization at a rate of 1 in 250 and that men reported significantly higher rates of CSA by these perpetrators compared to women.

CSA, in an institutional context also occurs through boys engagement with sport, which afford the opportunity, the environment, and a particular culture; “These organizations, particularly sports teams, may also provide the opportunity for CSA by taking children away from home (e.g., away games), normalizing nudity (e.g., locker rooms), and encouraging boys to act manly through expressions of bravery, aggression, and risk-taking” (Coburn et al., 2019, p. 600). The tight team environment of sport and recreation groups, where the team comes first, and there is an expectation of team solidarity may inhibit disclosure (Wolfe et al., 2003). There is some evidence that peer perpetrated abuse is significant in the sports sector, with one study (Pankowiak et al., 2023) finding that the rate of peer on peer violence was at 69%.

A common pattern has been documented (Burmester, 2018; Caroll et al., 2015; Driscoll, 2021) of institutions protecting perpetrators, ignoring and failing to follow up on complaints and allowing perpetrators to remain in organizations, sometimes at different locations, thus sanctioning continued access to, and abuse of children. This pattern of protection has meant numerous institutions became havens for pedophiles (Etherington, 2000). By the mid-2010s, approximately 20 government inquiries into institutional child abuse had been established around the world, in Nordic and Western European countries, Canada, the United States, Australia, and New Zealand (Swain, 2018). These inquiries have resulted in the implementation of child safeguarding policies and practices, including increasing regulatory oversight of institutions.

### Impact of CSA: Gender Considerations

An early review of studies of the long-term effects of CSA, found “. . . depression and self-destructive behavior, anxiety, feelings of isolation and stigma, poor self-esteem, difficulty trusting others, a tendency toward revictimization, substance abuse, and sexual maladjustment” (Browne & Finkelhor, 1986, p. 66). Male victim/survivors commonly suffer shame, guilt, and self-blame (Dorahy & Clearwater, 2012; Hunter et al., 1993; Lateef et al., 2023; Romano & De Luca, 2001). A study of male victim/survivors of clergy perpetrated CSA in the Catholic church (John Jay College of Criminal Justice, 2004)^3^ found individuals commonly suffering with post-traumatic stress disorder (PTSD) as well as relationship problems and substance abuse. Additionally, there was a change to victim/survivors’ religiosity and spirituality. Other studies confirm CSA is strongly associated with PTSD and alcohol and substance abuse (Alaggia & Millington, 2008; Butt et al., 2011; Halpern et al., 2018; Scott et al., 2023).

There is evidence that male survivors of CSA are significantly impacted throughout their lives (Van Roode, 2009), including their social and sexual functioning, emotional, and psychological well-being (Easton, 2014; O’Leary et al., 2017). Cashmore and Shackel (2014) found CSA of boys is distinguished by the use of violence and threats of physical harm and the presence of multiple abusers. Men who have been sexually abused as a child are 10 times more likely than men in the general population to have a clinical diagnosis (O’Leary & Gould, 2009), to suffer with more debilitating mental health outcomes (Spataro et al., 2004), and to have more emotional and behavioral problems. These men report suicidal ideation at a significantly higher rates (O’Leary & Gould, 2009) and have higher risk of attempting suicide (Garnefski & Arends, 1998; Garnefski & Diekstra, 1997; Independent Inquiry into Child Sexual Abuse, 2017; Martin, 2004; Molnar et al., 2001).

### CSA: Disclosure and Prevalence

Researchers suggest studies of CSA underestimate prevalence (Stoltenborgh et al., 2011) and acknowledge “a high *dark figure* of unreported offenses” (Böhm et al., 2014, p. 648). Studies estimating prevalence of male CSA vary: 14% (Briere & Elliott, 2003), 16% (Dube et al., 2005), and 17% (Lisak, 1995) with a meta-analysis of 55 studies worldwide on CSA prevalence finding the rate at between 3% and 17% (Barth et al., 2013). A recent study found CSA rates for males at 18.8% or one in five (Haslam et al., 2023).

Several factors may contribute to males, not disclosing, deferring, or delaying disclosure. Research attributes this to socially defined gender roles—the norms of masculinity—developed and reinforced from childhood to adulthood (Dhaliwal et al., 1996; Easton, 2014; Kia-Keating et al., 2005; Lisak, 1994; Romano & De Luca, 2001; Tang et al., 2008). Gender role socialization inform the male understanding of what being a man means—*strong, self-reliant, not a victim*. These masculine norms are instrumental in male disclosure/non-disclosure decisions as well as help-seeking (Seidler et al., 2016). Men often report that *shame* is commonly a barrier to seeking treatment (Rapsey et al., 2020). Delayed disclosure and help-seeking can prolong and exacerbate physical and mental ill health and lead to protracted recovery.

Research highlights male victim/survivors fear they will be viewed as homosexual or weak (Cermak & Molidor, 1996; Collin-Vézina, 2015; Dorahy & Clearwater, 2012; Finkelhor, 1979; Hunter, 1991) and others are concerned they will be perceived as at risk of sexual offending (Dhaliwal et al., 1996; Royal Commission into Institutional Responses into Child Sexual Abuse, 2017b). *Being threatened* by the perpetrator also factors in disclosure/non-disclosure (Royal Commission into Institutional Responses into Child Sexual Abuse, 2017a). Other victim/survivors report being confused; not understanding and not being able to articulate what has occurred (Alaggia, 2004; Easton, 2014; Kia-Keating et al., 2005). Victim/survivors may have been told by their abuser that this is a normal part of their childhood sexual experiences/sexual development or minimized the abuse as sexual exploration or experimentation (Cashmore & Shackel, 2014; G.Holmes, 1997; W.C.Holmes, 2008; Hunter, 1991).

There are significant limitations of the knowledge base on institutional CSA, one substantial gap is the inadequate focus on male survivors, despite strong evidence of high prevalence. A strong evidence base attests to the negative impact of institutional CSA on males over the life course. This has important implications for conducting this scoping study which will consider the emerging evidence, to consider “. . . the extent, range and nature of research activity” (Arksey & O’Malley, 2005, p. 21), to summarize the research findings (Peters, 2015), and to identify gaps in the research literature (Arksey & O’Malley, 2005).

## Method

Scoping reviews have the advantage of being systematic, transparent, and replicable (Grant & Booth, 2009). Scoping review methodology was used in this study to systematically explore and provide an overview of the literature. The review followed the Arksey and O’Malley (2005) framework of five-stages: (1) identifying the research question; (2) identifying relevant studies; (3) study selection; (4) charting the data; and (5) collating, summarizing, and reporting results.

The search strategy for the review was to identify English language peer reviewed research and relevant reports, from 2000 to 2023, that engaged adult male survivors regarding their experience of institutional CSA. The terms used in the searches were refined in discussion with the research team and included: child sexual abuse or child maltreatment; males or men or man or boy or youth or adolescent; institution or church or priest or cleric or clergy or coach or sport or recreation or scout or school or education or foster or childcare or armed forces or defense or defence or military or navy or youth detention or juvenile justice or hospital or healthcare.

Five databases were searched—APA PsycINFO, CINAL, Medline, Scopus, Web of Science—and 973 studies were identified. A search of gray literature using the Google Advanced Search resulted in 96 additional references. Once relevant studies were identified, a “snowballing” exercise was undertaken—reference scanning relevant studies—which revealed 14 further studies. One hundred eighty-four full-text articles were assessed for eligibility. The appraisal at this stage considered the setting rationale, appropriateness of the sample, adequacy of the description of fieldwork, and adequate evidence to support analysis (Long & Godfrey, 2004).

The first search for literature was undertaken by the primary author (P.W.). Another author (M.T.) assisted in reviewing and providing advice on studies which were considered borderline for inclusion. This allowed the authors to be specific and clear regarding inclusion/exclusion criteria. Three of the authors (P.O’L., M.T., and A.Y.) are experienced academics and researchers who appraised selected articles and provided direction, guidance, and reviews of drafts.

To concentrate the selection of studies on research on adult male survivors who had experienced institutional CSA, a decision was made to exclude prevalence studies of CSA, meta-analyses, and systematic reviews. The criteria for inclusion were studies where 50% or more of the participants were adult males; studies which identified 50% or more participants had institutional perpetrators; and studies which had a focus on or had delineated CSA from other forms of child maltreatment.

Excluded from this research were reviews of studies, data linkage studies, research on complaints or reports of CSA, and validation of assessment tools. Also excluded was research focused on female victim/survivors, males 16 years old and under, studies which primarily focused on victim/survivors of familial CSA, physical and emotional abuse, neglect, and the sexual assault of adults.

**Figure 1.**
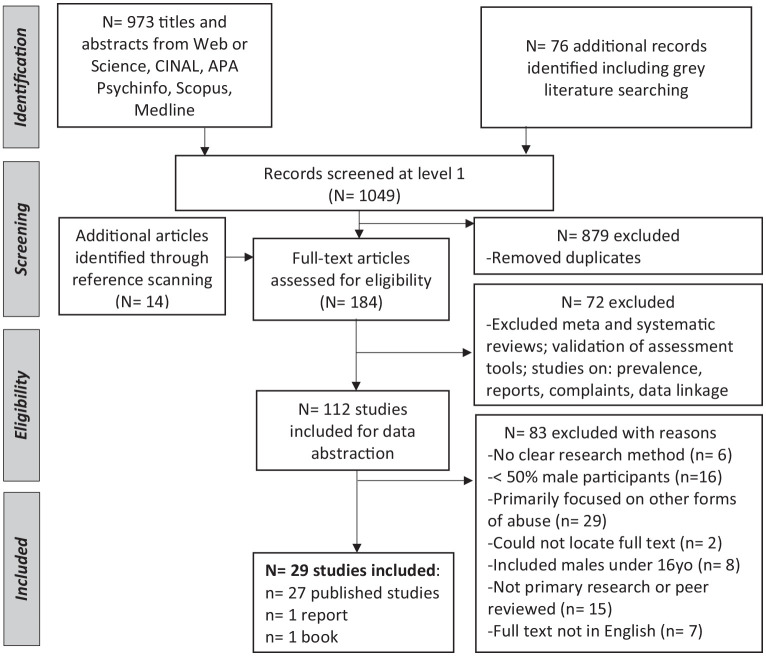
The study flow, including the number of records and reasons for inclusion and exclusion.

The search and analysis (Figure 1) followed the Preferred Reporting Items for Systematic Reviews and Meta-analyses (PRISMA) guidelines and the improved model for scoping reviews (Tricco et al., 2016).

Articles were reviewed many times to ensure relevance and alignment with the study’s purpose and the research question (Levac et al., 2010) and through the review and reflection on the 29 identified studies, themes became clearer, with studies clustered into themes including disclosure, survivor experience and impact. A template was developed to assist in the sorting of data into themes and issues (Arksey & O’Malley, 2005). This allowed analysis within these themes and an ability to compare and contrast across the identified studies. In order to analyze and discuss findings of methodologically diverse studies a tabular synthesis with narrative commentary is utilized (Grant & Booth, 2009).

## Findings

Twenty-nine studies were identified that had some focus on institutional CSA of males. Each of the selected studies varied in aspects of their research focus. Table 1, in the column headed *Aim/Focus*, shows the categories of research focus against each study: survivor experience (*n* = 9), impact (*n* = 13), disclosure (*n* = 5), turning points (*n* = 1), and meaning making (*n* = 1). Studies which had a focus on *disclosure* or *impact* were easily identifiable, whereas the category *survivor experience* covers a range of research studies such as survivors of institutional CSA being asked about what they consider helpful institutional responses (Blunden et al., 2021), and comparison of institutional CSA experienced at three types of institutions: Roman Catholic, Protestant, and non-religious (Sprober et al., 2014). Two additional studies were included: one which examined turning points (Easton et al., 2015), and the other which considered meaning making (Krinkin et al., 2022). The studies in the *survivor experience* group frequently include some content on other relevant issues. Following analysis, the decision was made to report the primary focus of the selected research papers under the following headings: disclosure, impacts (emotional, mental health, alcohol, and other impacts), and what is helpful to victim/survivors.

**Table 1.** Studies of Institutional CSA of Males.

Author(s), Date Country Journal	Participants	Institution/Perpetrator	Method	Aim/Focus	Identified Themes/Main Findings
1. Balboni and Bishop (2010) U.S. *Contemporary Justice Review*	22 • 15 male • 7 female	Catholic clergy	In-depth interviews	Survivor experience Exploring the experience of survivor—litigants, in their litigation of the Catholic Archdiocese of Boston	• Survivors felt alienated from the Church and expressed that their trust had been betrayed. • Survivors’ motivation for litigation was wanting to be believed and acknowledged. • Many survivors wanted a sincere apology from the church.
2. Blunden et al. (2021) Australia *Journal of Child Sexual Abuse*	8 • 5 male • 3 female	Various institutions • Including schools, boarding schools, gymnasiums, youth detention, armed forces	Thematic analysis of in-depth interviews	Survivor experience Survivors of institutional CSA were asked about what they consider helpful institutional responses	• Survivors interactions with institutions where they had been sexually abused as children were largely negative. • Positive responses from institutions looked like: acknowledgment of responsibility; a meaningful apology; protection of other children/prevention of further abuse; taking action against the perpetrator, including criminal justice; accountability and transparency; redress including compensation; counseling or support.
3. Carr (2009) Ireland *Attachment & Human Development*	247 • 54.7% male (*n* = 135) • 45.3% female	Religiously affiliated reformatories and industrial schools	Interviews, Inventories, and Scales	Impact Examines survivors of institutional care, adult attachment styles	• More than half of the participants reported they had experienced sexual abuse. • “Adult survivors of institutional abuse with fearful adult attachment styles had more psychopathology and showed poorer psychosocial adjustment in terms of quality of life, global functioning, marital satisfaction, and marital stability” (p. 198).
4. Carr et al. (2010) Ireland *Child Abuse & Neglect*	247 • 135 male • 112 female	Various institutions • 49% in institutions managed by nuns; 31% in institutions managed by male religious	Interviews, Questionnaires, Inventories	Impact Adult adjustment and mental health of survivors of institutional CSA	• The prevalence of psychological disorders among adult survivors was over 80%. • A comparison long-term impacts of institutional CSA of males and females found males had a higher likelihood of lifetime alcohol dependence. • Most survivors experienced PTSD symptoms and insecure attachment.
5. Carr et al. (2019) Scotland *Child Abuse & Neglect*	225 • 149 male • 76 female	Various religious and non-religious foster and residential care institutions	Thematic analysis of witness statements to the Scottish Child Abuse Inquiry	Impact The long-term impact (mental and physical health and psychosocial adjustment) on survivors of institutional abuse	• Sexual abuse was reported by 60% of the participants and the more severe the abuse the greater the physical and mental health impacts and adjustment issues. • The most common psychosocial adjustment difficulties were career development problems, and problems trusting other people. • Most survivors reported protective factors such as supportive relationships and skill development.
6. Colton et al. (2002) U.K. *British Journal of Social Work*	24 • 22 male • 2 female	Residential homes	Interviews	Survivor experience Reflections on the experience of victims/survivors involved in investigations of CSA in residential institutions	• There has been a historical indifference to children in care and a lack of child rights focus and the legal system can often discredit survivors through the stigma of being children in care. • For some survivors a public apology might be more welcomed and therapeutic than financial redress. • Many survivors recounted the barriers to disclosing abuse while still in care. • Survivors concerned about being perceived as potential child abusers.
7. Corrado and Cohen (2003) Canada *The Aboriginal Healing Foundation Research Series*	127 • 70% male (*n* = 89) • 30% female	Residential schools largely run by Christian religious orders	Case file analysis—coding of psychological assessments then statistical analysis	Survivor experience Understanding the complex aspects of mental health issues for those who had been placed in residential schools	• 100% of subjects reported sexual abuse and 90% reported physical abuse. • Mental health concerns in three-quarters of subjects, primarily PTSD, substance abuse disorder and major depression. • Over three-quarters of the subjects had abused alcohol after leaving the residential school. • Half the subjects had criminal histories mainly involving assault and sexual assault. • One fifth of the sample were expelled from schools or have quit.
8. Easton (2013) U.S. *Clinical Social Work Journal*	487 males	Various institutions • 82% identified as survivors of clerical abuse	Survey and Scales with purposive sampling	Disclosure Identifying disclosure processes and differences, disclosure and mental health	• Men delay *telling* on average by 21 years and *discussing* the abuse by 28 years. • Nearly all participants had told someone about the abuse at some point. • The mean age at the time of first disclosing was 32 years old. • Half the participants first told a spouse/partner or a mental health professional.
9. Easton (2014) U.S. *Child Abuse & Neglect*	487 males	Various institutions • 62% of participants abused by clergy	Anonymous internet-based survey, including Scales, Indexes, and Inventories. Cross-sectional survey design with purposive sampling.	Disclosure Explores factors related to disclosure and mental distress in male survivors	• Men often had unhelpful responses when disclosing. • Men sought out individuals who could provide support and respond with help and encouragement. • Both the type and source of support are important considerations in recovery. • There are no significant differences, in terms of mental distress, between male survivors abused by clergy and those abused by non-clergy.
10. Easton et al. (2019) U.S. *Journal of Interpersonal Violence*	205 males	Religious institutions • Participants identified their abuser as a member of the clergy—a priest or nun	Survey and qualitative analysis, secondary analysis of data collected during a 2010 Health and Well-Being Survey (Easton, 2014)	Impact The impact of CPSA on survivors’ long-term self-identity	• Most participants they identified their abuser as male (98.5%) and abuse lasted for more than 6 month (67.8%). More than half indicated the abuse involved penetration and one fifth indicated they were physically injured by the sexual abuse. • The findings support existing research which indicates the impacts of clergy perpetrated CSA on survivors are lifelong.
11. Easton et al. (2015) U.S. *Journal of Child Sexual Abuse*	250 males	Various institutions • In over half the cases the abuser was a member of the clergy	Survey and qualitative analysis, secondary analysis of data collected during the 2010 Health and Well-Being Survey (Easton, 2014)	Turning points Examines types of turning points experienced by male survivors	• Male survivors reported their abuser was male (95%) and in over half the cases (57%) the abuse involved penetration. In 57% of cases the abuser was a member of the clergy and in 7% was a teacher or a coach. • “. . . researchers have found that receiving a supportive response to disclosure of CSA is related to better mental health outcomes for adult survivors” (p. 168).
12. Easton and Parchment (2021) U.S. *Child Abuse & Neglect*	487 males	Various Institutions • 61.6% abuse by clergy • 6.8% abuse by teacher of coach	Online survey—qualitative content and inductive thematic analysis	Disclosure The characteristics of a helpful response to male survivors	• Key characteristics of helpful responses include listening and believing survivors; being empathic, validating and supporting help seeking. • For 59% of men the person who was most helpful to them when discussing the abuse was not a professional but a family member or friend or fellow survivor.
13. Easton et al. (2014) U.S. *Psychology of men & masculinity*	460 males	Various institutions • 61.7% abuse by clergy • 6.8 % abuse by teacher or coach	Online survey—content analysis of secondary data	Disclosure To identify barriers to disclosure	• Disclosure identified as multifactorial—a complex and extended process. • Disclosure generally proceeds help seeking and therefore it is important to ensure resources and referral. • Two-thirds of participants were abused by clergy. Research suggests that there may be different disclosure barriers for male survivors of clergy perpetrated CSA.
14. Elias et al. (2012) Canada *Social Science & Medicine*	2,953 • 55% male (*n* = 1,624) • 45% female	Residential schools largely run by Christian religious orders. Of the total participants: • 611 attended and 2,342 did not attend, residential schools • 1,100 were offspring of residential school attendees	Interview data from the Manitoba First Nation Regional Longitudinal Adult Health Survey	Impact Exploring potential predictors of a lifetime history of abuse, suicidal thoughts and suicide attempts—comparing residential and non-residential attendees and the children of survivors	• The trauma of institutional abuse may mean victim/survivors cope ineffectively as they age and there are potential similarities between this cohort and the experience of aging holocaust survivors. • Cultural experience and indigenous perspectives are important in understanding and responding to trauma. • Suicide thoughts and attempts are associated with having a parent or grandparent who attended a residential school.
15. Fater (2000) U.S. *Issues in Mental Health Nursing*	7 males	Clergy abuse • Catholic and Episcopalian	Semi structured interviews and use of phenomenological method of data analysis	Survivor experience The lived experience, focused on emotional impact, of male survivors of CSA by clergy	• Male survivors reported they were angry, guilty, ashamed and fearful. • Confusion is another emotion which was common among survivors, this often resulted in difficulty in everyday life. • Survivors’ embarrassment and shame contributes to the tendency to not disclose—this suits church leadership who can continue to cover up and deny the problem.
16. Fitzpatrick et al. (2010) Ireland *Child Abuse Review*	247 total • 54.7% male (*n* = 135) • 45.3% female	Religiously affiliated reformatories and industrial schools	Interviews, including questionnaires and inventories, scales, and checklists	Impact Examining the profiles—sexual, physical and emotional—of those who have experienced severe institutional abuse	• The group who had experienced severe sexual abuse had also experienced severe physical abuse. • This group had spent more time with their families prior to institutional placement and were often placed in care due to their involvement in petty crime. • “Survivors of severe sexual abuse had the most abnormal profile, which was characterized by higher rates of all forms of child maltreatment and higher rates of PTSD, alcohol and substance abuse, antisocial personality disorder, trauma symptoms and life problems” (p. 387).
17. Isely et al. (2008) U.S. *Journal of Child Sexual Abuse*	9 males	Catholic clergy	In-depth interviews used in qualitative analysis	Impact The impact of CSA on men abused by Catholic clergy	• All except one victim/survivor did not disclose immediately for fear they would be punished and not believed. • Reflecting on their experience victim/survivors did not believe at the time what they experienced was abuse or illegal. • All men in the study reported the long-term impact of clergy perpetrated CSA, including, intrusive memories with some reporting flashbacks as adults. • Male survivors reported anger, shame, guilt and confusion as well as mood and sleep disturbance and suicidal ideation.
18. Katz et al. (2017) Australia *Social Policy Research Centre University of NSW*	61 • 42 male • 19 female	Various institutions that provide/provided activities, facilities, programs or services through which adults have contact with children	Analysis of transcripts from the Royal Commission into Institutional Responses to Child Sexual Abuse	Survivor experience To understand the experience of survivors over the life course	• Most victim/survivors received negative responses when they reported to institutions seeking help. • A key positive outcome identified by survivors was having a supportive partner. • Therapy and counseling was identified by survivors as being generally helpful—noting it was important to find the right counselor. • Most victim/survivors showed some resilience in, despite their difficulties they had experienced they had overcome these, even though they continued to experience ongoing social/psychological problems.
19. Krinkin et al. (2022) Israel *Child Abuse & Neglect*	8 males	Jewish rabbis	In-depth semi-structured interviews	Meaning making Consider the implication of rabbi CSA on victim/survivors (Israeli still religious men)	• Half the participants noted a *religious weakening* • The study supports the meaning-making coping model. • “These findings which point to a decline in religiosity among half the participants, seem to indicate that the ability of CPSA victims to use religious coping strategies may have been impaired” (p. 9).
20. Lueger-Schuster (2014) Austria *Child Abuse & Neglect*	448 • 340 male • 109 female	Survivors of institutional abuse connected to the Catholic Church	Interviews and questionnaires	Impact Examination of types of abuse and mental health impact	• Most victim/survivors experienced at least two types of abuse, with child emotional abuse being the most prevalent. • CSA was reported by 68.8% of participants and physical abuse was reported similarly (68.3%). • Half of the participants had PTSD and participants who experienced CSA involving penetration had more severe mental health consequences.
21. Lueger-Schuster et al. (2018) Austria *Child Abuse & Neglect*	220 • 132 male • 88 female	Various institutions—institutional foster care	Questionnaires, Inventories, and structured clinical interviews. Additionally, this research engaged a control group (*n* = 234) from the Viennese population for comparison.	Impact Lifetime impact of child abuse and neglect in foster care	• Mood disorders and substance use disorders were consistently reported by participants, impacting them across their life course. • This cohort of victim/survivors were found to have a high prevalence of depression, PTSD and personality disorders. • Those in foster care have a significantly higher prevalence of mental illness and PTSD symptoms than the comparison population who have not been in care.
22. Lusky-Weisrose (2021) Israel *Child Abuse & Neglect*	21 males	Jewish institutions • CSA by authority figures in ultra-Orthodox Jewish institutions	In-depth interviews	Survivor experience Focus is on perceptions of survivors and their relationships with their abusers	• Participants, as children, did not understand that what had occurred was abuse and their understanding shifted over time and in adulthood to recognize it as CSA and its impacts. • Initially victims perceived their relationship with the perpetrator as: normative; mutual; routine; punitive; or severe sexual abuse. • Most participants expressed guilt or shame and many even believed themselves to be sinners.
23. Pereda et al. (2022) Spain & Chile *Journal of Child Sexual Abuse*	40 • 29 male • 11 female	Catholic clergy	Interviews involving 182 males and females and considered three groups: 1. the clergy; 2. the family; 3. external to clergy and family	Impact A study on the impact, in terms of mental health, social and spiritual consequences, on victims of CSA by Clergy and others.	• The effects of clergy perpetrated CSA often leads to a decline in belief in God and predicts negative social and mental problems. • This study found the impacts of CSA were similar in cases of intrafamilial and clergy perpetrated abuse. • “. . . the dynamics of institutional CSA may be better described by the term *entrapment*” (p. 404).
24. Ponton and Goldstein (2004) U.S. *Adolescent Psychiatry*	26 males	Catholic clergy	Diagnostic evaluation interviews and data evaluation	Impact Considers the length of time it takes to report, the characteristics of priests, psychological sequelae, spiritual and sexual concerns	• Males took 18 years or more before seeking help. • Of the 26 men in the study, 85% met the DSM criteria for depression and 88% met the criteria for substance abuse. Over half the men reported impacts including suicidality, loss of spirituality and sexual issues. • Researchers conclude both physical and sexual abuse is under-reported by adolescent boys. • CSA prior to 13 years old was associated with more profound psychological symptoms.
25. Shea (2008) U.S. *Sexual Addiction & Compulsivity*	49 males of which 29 had experienced CPCSA	Clergy perpetrated CSA • Catholic priests and brothers	Questionnaires including inventories and scales	Impact The effects of clergy perpetrated CSA on adult survivors	• Both the group who experienced clergy perpetrated CSA and those who experienced CSA by another, reported significant negative effects with no differences in impact between the two groups. • Those abused by clergy did tend to hold off disclosing until they were older. • There are clear sociological factors, for example, stigma, fear of becoming or being perceived as homosexual, which contribute to underreporting of male CSA. Some of these factors appear to be reinforced by the institutional church.
26. Sheridan and Carr (2020) Ireland *Child Abuse & Neglect*	9 • 5 male • 4 female	Various institutions • Survivors of historical institutional abuse from orphanages, industrial schools, Magdalene laundries.	Semi-structured interviews with data analyzed—IPA	Survivor experience To explore positive change of adult survivors of institutional childhood abuse	• An *important other* contributing to positive change was reported by all participants. • Relationship issues, family conflict, domestic and family violence, and relationship breakdown were reported by many participants. • Survivors felt marginalized and stigmatized by their early life experiences of being in an institution. • PTSD symptoms were commonly reported by participants, including intrusive memories and re-experiencing abuse.
27. Sprober et al. (2014) Germany *BMC Public Health*	1,050 59.8% males (*n* = 628)	Various institutions • Roman Catholic (*n* = 404) • Protestant (*n* = 130) • Non-religious (*n* = 516)	Anonymous information provided to government established hotline for victims—information was documented and categorized, and descriptive analysis applied	Survivor experience Comparison of institutional CSA experienced at three types of institutions: Roman Catholic, Protestant, and non-religious	• Religiously affiliated institutions cultures and structures, along with a neglect of children’s rights contributed to the historical prevalence of CSA. • Patterns of abuse and gender of offenders did not differ, and the mental health impacts were similar across the three groups (Catholic, Protestant, and non-religious). • Approximately half the victims had been physically and sexually abused. • Most reported repeated abuse was committed by male perpetrators.
28. Wolfe et al. (2006) Canada *Child Abuse & Neglect*	76 males	Religiously affiliated institutions	Clinical interview, diagnostic interview, psychological tests	Impacts To describe the long-term impacts of physical and sexual abuse on males, whereby the perpetrator is outside the family in a religious institution	• Participants met the criteria for PTSD (42%), alcohol abuse (21%), and mood-related disorders (25%). • A third of participants suffered with sexual problems, and a half had a criminal history. • Participants noted the challenges experienced trying to disclose often because “. . . the institution was located in a small, closely knit community that was bound by cultural, ethnic, and religious identities . . . formidable resistance still remains to acknowledging the men’s abusive experiences and ongoing needs” (p. 209).
29. Zalcberg (2017) Israel *Journal of Child Sexual Abuse*	40 males	Jewish institutions	Ultra-Orthodox Jewish (Haredi) men. In-depth interviews and qualitative analysis based on grounded theory	Disclosure Reporting patterns of sexual abuse of males in a specific religious-cultural context	• Some men in this study did not realize until their adulthood that what they had experienced was CSA. • Barriers to reporting included *it* being considered as profanity and not being able to be discussed with parents, embarrassment, shame and guilt, and a concern about being blamed. Others reported they did not want to cause their parents’ distress. • There is a silencing of issues in relation to sexuality and a tendency to cover up incidents of CSA possibly influenced by these issues being considered taboo and sinful in conservative religious communities.

*Note.* CSA = child sexual abuse; CPSA = clergy perpetrated sexual abuse; IPA = interpretive phenomenological analysis; PTSD = post-traumatic stress disorder.

Twenty-four studies had a sole focus on survivors of institutional abuse, that is, they were not considering abuse outside of institutions. Fourteen studies involved only male participants. The types of institutions/perpetrators examined by the studies (where stated in the study) included: Catholic, Episcopalian, Jewish, Protestant, and non-religious institutions; schools, boarding school, gymnasiums, sports, youth detention, armed forces, reformatory and industrial schools, orphanages, foster care and residential care, residential schools, and Magdalene laundries. Studies came from Australia (*n* = 2), Austria (*n* = 2), Canada (*n* = 3), Germany (*n* = 1), Ireland (*n* = 4), Israel (*n* = 3), Scotland (*n* = 1), Spain and Chile (*n* = 1), the United Kingdom (*n* = 1), and the United States (*n* = 11).

Several researchers have multiple articles in this scoping study. Six of the studies have Easton as lead author (Easton, 2013, 2014; Easton & Parchment, 2021; Easton et al., 2014, 2015, 2019); Carr is the lead author on three studies (Carr, 2009; Carr et al., 2010, 2019) and an author on another two (Fitzpatrick et al., 2010; Sheridan & Carr, 2020); and, Lueger-Schuster is lead author in two studies (Lueger-Schuster, 2014; Lueger-Schuster et al., 2018). The concentration of a few researchers in this field, coupled with the limited number of overall studies illustrates the relatively minor attention on this area. Twenty-seven studies are from peer reviewed journals. One study is a report, and another is a book. All studies were published between 2000 and 2023 in English.

Seven studies were focused on abuse by Catholic Clergy (Balboni & Bishop, 2010; Fater, 2000; Isely et al., 2008; Lueger-Schuster, 2014; Pereda et al., 2022; Ponton & Goldstein, 2004; Shea, 2008). Three studies were focused on abuse through Jewish institutions (Krinkin et al., 2022; Lusky-Weisrose, 2021; Zalcberg, 2017). Seven studies indicated more than 50% of the victim/survivors involved in their research were abused by clergy (Easton, 2013, 2014; Easton & Parchment, 2021; Easton et al., 2014, 2015, 2019; Sprober et al., 2014). A further eight studies indicated the institutions were largely religiously affiliated (Carr, 2009; Carr et al., 2010, 2019; Corrado & Cohen, 2003; Elias et al., 2012; Fitzpatrick et al., 2010; Sheridan & Carr, 2020; Wolfe et al., 2006). This means that most studies, 25 of the 29, involved victim/survivors whose CSA resulted from religious institutions.

Research methods used in the selected studies include interviews (clinical, diagnostic, semi-structured, in-depth), use of psychological tests, diagnostic checklists, inventories and scales; questionnaires and surveys; transcript analysis, content analysis, case file analysis, and thematic analysis. A total of 6,097 males participated in the 29 research studies. The largest study (Elias et al., 2012), which examined interview data from the Manitoba First Nation Regional Longitudinal Adult Health Survey, included 1,624 males. Several small studies involved structured, in-depth interviews with less than 10 men who had experienced institutional abuse, including: Blunden et al. (2021) 5 men; Fater (2000) 7 men; Isely et al. (2008) 9 men; Krinkin et al. (2022) 8 men; and Sheridan and Carr (2020) 5 men.

### Disclosure

Disclosure of CSA by men can relate to a number of factors including the burden of mental distress (Easton, 2014), concerns about being believed or being punished and, in some cases, not understanding that what occurred was abuse and therefore illegal (Isely et al., 2008). In Colton et al. (2002) survivors identified the barriers to disclosing abuse whilst still in *care*. Two studies of men who experienced CSA in Jewish institutions (Lusky-Weisrose, 2021; Zalcberg, 2017) found that, at the time, men did not recognize that what they experienced was abuse. Barriers to reporting included concerns about being blamed, not being able to discuss experience with parents, and the experience being considered forbidden and sinful. Illustrating this point about the barriers to disclosure, the Zalcberg (2017) research reports that, of the 40 male participants in the study, 65% had not disclosed their CSA until the interview with the researcher.

Research on disclosure and help seeking by male victim/survivors of CSA has found men can take, on average, between 18 years (Ponton & Goldstein, 2004) and 21 years (Easton, 2013). Deciding who to speak with is a critical decision for survivors (Balboni & Bishop, 2010). Easton (2013) found the mean age for men to disclose was 32 years old; half the 487 male survivors in his study first told a spouse/partner.

### Impact

Most of the 29 studies addressed impact and leave no doubt that CSA of males results in significant and long-lasting impairment. Impacts have been clustered into three areas: emotional impact, mental health impact, and alcohol and other impacts.

The emotional impact was reported in several studies. Commonly, male survivors report feelings of embarrassment, guilt, intense shame (Lusky-Weisrose, 2021; Ponton & Goldstein, 2004; Zalcberg, 2017), fear (Fater, 2000), distress (Lueger-Schuster, 2014), self-blame (Lusky-Weisrose, 2021), confusion, sadness, and avoidance of relationships (Isely et al., 2008). Survivors have difficulty trusting people throughout their lives (Carr et al., 2019). Another emotion expressed by male survivors is that of anger (Fater, 2000; Isely et al., 2008), with male victim/survivors of clergy perpetrated CSA expressing anger not only at their abuse but also at the Church’s response to the abuse crisis—denial, coverup, and silence—with this anger being expressed externally as rage and also impacting survivors’ mental health.

Mental health impact was reported in numerous studies, including disorders related to mood, anxiety, depression, personality, post-traumatic stress (Isely et al., 2008; Lueger-Schuster et al., 2018; Wolfe et al., 2006) and suicidality (Ponton & Goldstein, 2004). PTSD is often accompanied by intrusive memories in institutional abuse survivors (Sheridan & Carr, 2020). In the Isely et al. (2008) study of men sexually abused as children by priests, “All reported symptoms of mood disturbance, such as low self-esteem, poor sleep, suicidal ideation, anger, and detachment from others following the abuse and intensifying in adulthood” (p. 207).

Three studies (Carr et al., 2010; Corrado & Cohen, 2003; Ponton & Goldstein, 2004) indicated the prevalence of depression or PTSD in more than three-quarters of institutional abuse survivors. Two factors appear to contribute to more severe mental health impacts: CSA prior to 13 years old (Ponton & Goldstein, 2004), and CSA with penetration (Lueger-Schuster, 2014).

In addition to the debilitating impacts of severe trauma and mental health symptoms, alcohol and other substance abuse impacts have also been identified (Fitzpatrick et al., 2010; Lueger-Schuster et al., 2018; Sheridan & Carr, 2020). Corrado and Cohen (2003) study of abuse survivors from Canadian residential schools found alcohol consumption was higher than the general population. Both Carr et al. (2010) and Wolfe et al. (2006) found a higher likelihood than the general population of lifetime alcohol dependence for male survivors of institutional CSA. Alcohol also played a role both in grooming where “. . . the priest gave the boy alcohol or drugs prior to the sexual abuse” (Ponton & Goldstein, 2004, p. 222), and subsequently impacted through lifelong substance abuse of many victim/survivors.

Other impacts include: being expelled or quitting school (Corrado & Cohen, 2003), difficulties with relationships, education and employment (Easton et al., 2019), general feelings of stigmatization and marginalization due to their abuse and institutional upbringing (Sheridan & Carr, 2020), a loss of spirituality (Ponton & Goldstein, 2004), a decline in belief in God and Church (Pereda et al., 2022), a religious weakening (Krinkin et al., 2022), sexual issues/problems (Ponton & Goldstein, 2004; Wolfe et al., 2006), and criminal behavior (Corrado & Cohen, 2003; Sheridan & Carr, 2020; Wolfe et al., 2006).

In considering the intergenerational impacts of the abuse from residential schools on the Canadian Indigenous population, Elias et al. (2012) found suicidal ideation and suicide attempts were associated with having a parent or grandparent with a history at a residential school.

### What Is Helpful?

In-depth interviews with survivors of institutional abuse in the United States (Balboni & Bishop, 2010) and Australia (Blunden et al. (2021), found survivors felt betrayed and alienated from a range of institutions where CSA had occurred and reported negative interactions when they tried to engage with the institution. Additionally, Balboni and Bishop (2010) found survivors of clergy perpetrated CSA wanted to support other survivors, and that they needed “. . . the larger community to bear witness to the wrongs perpetrated by the Church in order to vindicate survivors in the eyes of the community” (p. 147).

Easton and Parchment (2021) found that most victim/survivors reported it was helpful to discuss the abuse with a family member, or another survivor, where helpful responses involved “. . . listening and believing survivors’ CSA narrative, validating feelings, demonstrating empathy, and encouraging help seeking” (p. 8). Importantly for survivors, it is helpful for them to understand that they were not alone.

A study of survivors (Carr et al., 2019) of various foster and residential care institutions in Scotland found protective factors such as skill development and relationships that are supportive were valued by survivors. Sheridan and Carr (2020) study participants—survivors of historical institutional abuse in Ireland—expressed the significance of *an important other* in making positive change following CSA. Additionally, their research identifies the meaningfulness of turning points which can relate to post-traumatic growth.

Turning points and trigger events were mentioned in a number of the studies, with maturity and development over the life course presenting opportunities for change and more positive directions (Sheridan & Carr, 2020). Lusky-Weisrose (2021) found survivors acknowledge turning points following a variety of life events, with many indicating that insight was not sudden but emerged over time. While Katz et al. (2017) found trigger events were significant for survivors, these events could impact survivors negatively as well as positively; he also identifies the importance of a supportive partner and finding the right therapist/counselor. Easton et al. (2015) highlight three turning points from research with male survivors: influential relationships; insights and new meanings; and action-oriented communication, which can be helpful in supporting men and moving them toward healing. The Krinkin et al. (2022) study supports meaning-making as a coping model for victim/survivors.

Two key implications from this research are the need to increase education and training of health professionals regarding the understanding and impacts of institutional CSA of males and the need for institutions to consider research regarding victim/survivors in order to improve their responses. Both Table 2 Critical Findings, and Table 3 Implications for Practice, Policy, and Research, summarize the research findings.

## Discussion

This review assessed the research literature related to the institutional CSA of males. Few articles examined specifically focused on institutional CSA of males; among several of those that did, it was often part of a broader study of historical child maltreatment, or it included males and females and non-institutional forms of abuse. Given these limitations and the wide range of research methodologies used, drawing firm conclusions from the selected studies is challenging. Nevertheless, the studies paint a largely consistent picture of institutional CSA of males characterized by male perpetrators (Easton et al., 2015, 2019; Sprober et al., 2014) and with other forms of abuse, particularly physical abuse, accompanying sexual abuse (Carr et al., 2019; Corrado & Cohen, 2003; Fitzpatrick et al., 2010; Sprober et al., 2014). This abuse frequently involved penetration (Easton et al., 2015, 2019), multiple incidents (Easton et al., 2019), multiple perpetrators (Carr et al., 2019), and lasted over months or years (Easton et al., 2019).

The findings from the current review, such as the presence of violence or threatened force being linked to greater psychological distress (Fitzpatrick et al., 2010; Lueger-Schuster, 2014; Wolfe et al., 2006), are consistent with the literature (Cashmore & Shackel, 2014). The finding regarding the use of multiple forms of abuse is consistent with Haslam et al. (2023) whose study found one in four, or 25.4%, of victim/survivors experience three to five types of abuse and Ressel et al. (2018) findings “Males reported experiencing nearly three other types of maltreatment in addition to CSA” (p. 245). Other studies in this review (Carr et al., 2019; Easton, 2019; Isely et al., 2008; Lueger-Schuster et al., 2018) confirm negative impacts, notably mental ill health, over the life course. The impacts of institutional CSA of males coupled with men’s tendency to disclose abuse many years after the event, mean delayed disclosure (O’Leary & Barber, 2008) will likely have significantly impacted their mental health, relationships, and work over decades.

Findings from the current review also suggest males abused by clergy were older at the time the first abuse occurred and tended to report the abuse later (Shea, 2008). Some research (Easton, 2014; Shea, 2008; Sprober et al., 2014) indicates the impact of CSA appears to be similar whether perpetrated by clergy or another abuser; however, Pereda et al. (2022) found higher levels of psychological distress for victim/survivors of clergy perpetrated CSA.

Survivors have reported that their attempts to discuss/disclose their abuse were undermined by community support for the institution (Wolfe et al., 2006), and survivors and their families often consider that the Church has betrayed their trust (Balboni & Bishop, 2010). This betrayal of trust frequently results in victim/survivors losing their religion and not being able to use their belief in God/the church/the clergy to support them following their abuse (Krinkin et al., 2022; Pereda et al., 2022).

Institutional abuse impacted substantially on Indigenous children and children from disadvantaged backgrounds. These children are commonly the focus of state intervention resulting in institutionalization. The overrepresentation of these children is highlighted in the experience of Canada’s Indigenous population at residential schools (Corrado & Cohen, 2003; Elias et al., 2012; Wolfe et al., 2006) exposing the traumatic, long-lasting impacts on individuals, their families, and communities. There are parallels in Australia with Aboriginal and Torres Strait Islander children, who are more likely to be institutionalized (Tilbury, 2009) and overrepresented in institutional CSA (Royal Commission into Institutional Responses into Child Sexual Abuse, 2017b). Studies focused on intergenerational impacts, particularly for first nations populations, should be encouraged as they could usefully inform policy responses.

### Findings and Implications

**Table 2.** Critical Findings.

• There are limited empirical studies focused on male survivors of institutional CSA.
• Male victim/survivors of institutional CSA, as children and young people, often struggle to understand their abuse and as adults they can take some time to disclose, discuss, and seek help for the impacts of their abuse.
• Significant trauma results from CSA, with mental health impacts frequently lasting a lifetime.
• Alcohol and substance abuse in men commonly result from CSA.
• Victim/survivors want to be listened to and believed, a meaningful apology, to support other victim/survivors, and institutional reform.

*Note.* CSA = child sexual abuse.

**Table 3.** Implications for Practice, Policy, and Research.

Policy Implications
• Organizations need to implement child safety and well-being policies which are child centered and articulate child rights.
• Institutions need to consider research about victim/survivors regarding what is helpful for them and respond accordingly.
Research Implications
• Researchers need to involve victim/survivors in research to inform therapeutic treatment and gender-sensitive practice.
• There is a need for more longitudinal research for example; What does complex trauma in males look like over a lifetime? What are the inter-generational impacts of CSA of males on families and communities?
• Other consideration should be given to research on: older male victim/survivors; victim/survivors of female abusers; CSA by peers in institutional settings.
Practice Implications
• Increase education and training of mental health and drug and alcohol workers regarding institutional CSA of males, including screening for CSA.
• Treatments/interventions should be gender informed and sensitive.
• Increase referral and connection of victim/survivors, with allies, supports, and therapeutic services.

*Note.* CSA = child sexual abuse.

### Limitations

Only 29 studies were identified, and a smaller number had a sole focus on the CSA of males in an institutional context. A limitation in this research was one researcher (PW) completing the search. There were no studies identified for this review focused on specific youth serving organizations such as sports or scouts. Comparisons between studies was difficult due to different foci, aims, and methodology. Due to the different methods used, datasets were not quantitatively comparable. Non-peer-reviewed studies and studies not published in English were excluded from the review; this may have resulted in missing relevant research concerning the institutional CSA of males. It was, however, pleasing to include some studies in English from non-English speaking countries (*n* = 7). The three key search terms (and variations of these) “institutions,” “child sexual abuse,” and “males” clearly limited the scope, with several research articles meeting two, but not three, of these terms and therefore excluded.

There was variance in victim/survivor experiences both between and within studies. This is attributable to the different institutional context and type of sexual abuse, as well as the heterogeneous profile of most victim/survivor populations. Both results herein and broader literature identifies a range of factors (O’Leary, 2009) which may contribute to the variance of experience and outcomes such as the severity of the abuse; the number of perpetrators; the number of abuse incidents; age at time of the abuse; previous abuse; childhood circumstance; the institutions response; disclosure, help seeking; relationships and supports.

There remains an ambivalence by the public toward children in care (Colton et al., 2002). Often boys are over-represented in out-of-home care and youth detention. Two areas where little attention has been paid by researchers are male CSA in institutional contexts by peers and by female perpetrators. These continue to be areas of concern principally in the care system, education settings, and youth detention.

A final observation regarding limitations is the tendency in some of the literature and commentary to minimize or have less focus on male CSA, perhaps due to the lower prevalence rates for males and the gendered nature of CSA. This is despite the higher rate of male victims of institutional CSA and strong evidence that they are more likely to under-report CSA. The limited research in this area results in limited awareness, understanding, and ability to respond effectively to male survivors of CSA and, specifically, those where the abuse occurred in an institutional context. One wonders what other conditions/illnesses/diagnosis that account for up to one in five males would be considered such a low priority?

## Conclusion

This scoping review highlights the substantial, often hidden, long-lasting impact that institutional CSA has on men throughout their lives. Male victim/survivors of institutional CSA are a significant group who have been long overlooked, until recently. It is encouraging to see some research in this area particularly where it provides a better understanding of the impact of institutional CSA on males and what might be helpful in terms of recovery. However, there are significant gaps in the research literature and a need for increased research focus. Such research is critical in informing child safe environments, therapeutic, social, and justice supports for victim/survivors. More attention on the lived experience of men over the life course can contribute to justice for them and will assist researchers, families, professionals, and indeed institutions, in their responses to, and support of, victim/survivors. All of this urgently requires more investment in research, policy, and practice to improve the lives of male victim/survivors.

## Notes

1. The definition of CSA comes from the World Health Organization: The involvement of a child or an adolescent in sexual activity that he or she does not fully comprehend and is unable to give informed consent to, or for which the child or adolescent is not developmentally prepared and cannot give consent, or that violates the laws or social taboos of society (World Health Organization, 2017, p. vii).

2. The authors recognize a variety of terms are used to describe those who have experienced CSA. This article uses the term *victim/survivor*. The authors acknowledge that these terms are not without problems and mean different things to different people and that individuals will have preferences in terms of language is used.

3. The authors acknowledge the significant contribution this research (Terry, 2008; John Jay College of Criminal Justice, 2004) made to the understanding of clergy perpetrated CSA; however, the authors decided to exclude it from the selected studies in the scoping review. Although there was a victim survey as part of this research, it was completed by researchers “. . . based on information about the victim in the alleged abuser’s file” (John Jay College of Criminal Justice, 2004, p. 15) and was therefore excluded given the inclusion criterion was to hear the voices of victim/survivors in the research.
